# Experience with AMS 700 LGX penile prostheses for preserving penile length in Korea

**DOI:** 10.1186/s12894-018-0425-5

**Published:** 2019-01-16

**Authors:** Kang Sup Kim, Woong Jin Bae, Sae Woong Kim, Moo Yeon Lee

**Affiliations:** 1 Department of Urology, Incheon St. Mary’s Hospital, College of Medicine, The Catholic University of Korea, 59, Dongsu-ro, Bupyeong-gu, Incheon, 21431 Republic of Korea; 2 Department of Urology, Seoul St. Mary’s Hospital, College of Medicine, The Catholic University of Korea, 222, Banpo-daero, Seocho-gu, Seoul, 06591 Republic of Korea; 3 Catholic Integrative Medicine Research Institute, College of Medicine, The Catholic University of Korea, 222, Banpo-daero, Seocho-gu, Seoul, 06591 Republic of Korea; 4 Adams Urology Clinic, 513, Teheran-ro, Gangnam-gu, Seoul, 06169 Republic of Korea

**Keywords:** Erectile dysfunction, Penile prosthesis, Penile length

## Abstract

**Background:**

The purpose of this study was to report the outcomes of patients who underwent penile implantation with AMS 700 LGX inflatable penile prosthesis (IPP) in a single center by a single surgeon.

**Methods:**

A total of 342 patients with erectile dysfunction who underwent AMS 700 LGX IPP implantation between October 2014 and April 2016 were included in this study. All patients were evaluated using the International Index of Erectile Function questionnaire preoperatively and at 3, 6, and 12 months postoperatively. We also investigated the mean stretched flaccid penile length before and after surgery as well as the complications related to and the mechanical reliability of the IPP.

**Results:**

The questionnaire scores at 12 months were statistically significantly higher than the baseline scores. The mean stretched flaccid penile length was 11.1 ± 0.8 cm at baseline and was longer at 3 (11.9 ± 0.9 cm, *P* < 0.001), 6 (12.0 ± 0.9 cm, P < 0.001), and 12 (12.2 ± 0.7 cm, P < 0.001) postoperatively. There were no intraoperative or perioperative complications. However, one patient had infection and 10 patients developed mechanical failure during the follow-up duration.

**Conclusions:**

The results of our study suggest that the AMS 700 LGX IPP could be used to prevent penile shortening in patients undergoing IPP implantation. Furthermore, erectile function and patient satisfaction were improved excellently.

## Background

Although oral phosphodiesterase type 5 inhibitors, vacuum erection devices, and intracavernosal vasoactive agent injection are the preferred therapies for erectile dysfunction (ED), penile prosthetic implants are regarded as effective, safe, and durable for patients who are refractory to medical treatment and/or prefer a more effective and permanent therapy. Since Scott et al. [1] first reported using an inflatable penile prosthesis (IPP) in five patients, IPPs have been used for successfully treating ED for many years. Constant device improvements have resulted in good functional outcomes, with a low complication rate and high patient satisfaction. Patient dissatisfaction with penile girth occurs rarely. The penile length could be increased with time owing to the length expansion of the IPP. With a larger erect penis, one can have a longer flaccid penile length. However, patient satisfaction regarding penile length during erection with the prosthesis has not been achieved in some patients. Therefore, if patients are dissatisfied with their penile length postoperatively, daily inflation of the penile prosthesis is recommended to produce corporal expansion, followed by surgical replacement with a longer cylinder [2].

The American Medical Service (AMS) 700 Ultrex (American Medical Service, Minneapolis, MN, USA) was developed in the 1990s to promote penile lengthening. However, mechanical failure and complications occurred frequently; therefore, the use of this device was discontinued [3, 4]. AMS 700 LGX IPP (American Medical Service) was developed to overcome the mechanical problems of the AMS 700 Ultrex design to permit both penile girth and length expansion after IPP implantation. However, little has been reported about patient satisfaction postoperatively, especially with penile length, or the mechanical reliability or durability of this device, despite its widespread use by many andrologists [5].

## Methods

This study was aimed at evaluating and reporting the results of the use of AMS 700 LGX for IPP implantation, including preservation of penile length, complications (such as infection, hemorrhage, mechanical failure, or voiding problems), and patient satisfaction after surgery.

IPP implantation is performed in patients to treat ED if they do not respond to conventional ED therapy, including failure of oral phosphodiesterase type 5 inhibitors with maximal-dose or intracavernosal injection therapy. Patients who underwent implantation with the AMS 700 LGX IPP at a single andrology center between October 2014 and April 2016 were included. We excluded patients who were undergoing repeat implantation operation or prosthesis implantation because of Peyronie’s disease. Because ED treatment expenses, including IPP implantation procedures, are not covered by the Korean National Health Insurance program, the patient paid the related costs (operation and prosthesis fee and postoperative follow-up), and the type of penile prosthesis was selected by the patients and urologist together. Approximately 50% of the patients received AMS 700 LGX, 30% received AMS 700 CX, and 20% received AMS 700 CXR at the time of surgery (all from American Medical Service).

One andrologist (MYL) met all patients, gathered information on their medical history, and performed clinical and physical examinations. Data were obtained from the patients’ medical records and interviews. The data included early and late postoperative morbidities, such as infection, mechanical malfunction, and other surgical complications. Patients answered the International Index of Erectile Function (IIEF) questionnaire before undergoing surgery. When patients visited the outpatient department, they completed the questionnaire in private before meeting with an andrologist. The IIEF is a self-administered instrument comprising domains related to sexual function such as erectile function, orgasmic function, sexual desire, satisfaction with intercourse, and overall satisfaction [6]. The severity of ED was measured using the IIEF erectile function domain scores: < 10, severe ED; 11–17, moderate ED; 18–25, mild ED; > 25, no ED. The stretched flaccid penile length and penile length with the IPP fully inflated were also measured before IPP implantation and at 1, 3, 6, and 12 months postoperatively. The penile length was measured from the pubopenile skin junction to the penile meatus, along the dorsal side of the penile shaft.

We obtained approval from our institutional ethical review board to perform this study, which was conducted in accordance with the principles of the Declaration of Helsinki. Informed consent was obtained from patients before the IPP procedure.

All statistical analyses were conducted using the SPSS statistical software (SPSS Inc., Chicago, IL, USA). Since this is a retrospective study, sample size calculation was not needed. The preoperative and postoperative IIEF scores and stretched flaccid penile length were compared using Student’s t test. All data are presented as the mean ± standard deviation, and statistical significance was defined as *P* < 0.05.

## Results

A total of 342 patients underwent implantation with the AMS 700 LGX IPP between October 2014 and April 2016. The mean age of the patients and ED period were 58.3 ± 9.2 and 4.3 ± 2.5 years, respectively. None of the patients had previously undergone IPP implantation. The indications for IPP implantation were vascular ED for 134 patients, diabetes mellitus for 130, and pelvic surgery such as radical prostatectomy or cystectomy for 48 (Table 1).

**Table 1:** Patients’ demographics

Characteristic	Mean ± SD or n (%)
Age (years)	58.3 ± 9.2
ED duration (years)	4.3 ± 2.5
Cause of ED	
Vascular insufficiency	134 (39.2)
Diabetes mellitus	130 (38.0)
Pelvic surgery	48 (14.0)
Others	30 (8.8)

*ED* Erectile dysfunction

There were no perioperative or immediate postoperative complications. Transfusion and surgical revision were not required for any patients. Considering the long-term complications, one patient experienced infection at 3 months postoperatively and underwent IPP removal, and 10 patients experienced mechanical malfunction of the prosthesis.

The mean stretched flaccid penile length was 11.1 ± 0.8 cm at baseline and was longer at 3 months (11.9 ± 0.9 cm, *P* < 0.001), 6 months (12.0 ± 0.9 cm, P < 0.001) and 12 months (12.2 ± 0.7 cm, P < 0.001) postoperatively. There was no significant difference in the stretched flaccid penile length between 6 and 12 months (*P* = 0.31). The mean and penile length with the IPP fully inflated was 11.8 ± 1.2 at baseline, 12.6 ± 1.0 at 3 months, 12.6 ± 1.1 at 6 months, and 12.7 ± 1.2 at 12 months. There was a statistically significant difference in penile length with the IPP fully inflated from baseline to 3, 6, and 12 months (*P* < 0.001). No statistical difference in penile length with the IPP fully inflated was noted between 6 and 12 months (*P* = 0.14) (Table 2).

**Table 2:** Preoperative and postoperative penile length of the patients

	Baseline	Postoperative			*P*-value^a^
		3 months	6 months	12 months	
Flaccid penile length	11.1 ± 0.8	11.9 ± 0.9	12.0 ± 0.9	12.2 ± 0.7	< 0.001
Penile length with the IPP fully inflated	11.8 ± 1.2	12.6 ± 1.0	12.6 ± 1.1	12.7 ± 1.2	< 0.001

^a^Baseline flaccid penile length, penile length with the IPP fully inflated, and all of the follow-up period (postoperative 3, 6, and 12 months) penile lengths were compared, and a statistical significance was observed

Thirty-two of the 342 patients did not answer all of the questionnaires. Hence, 310 patients completed all of the questionnaires. We did not include this incomplete questionnaire in the present study. Table 3 presents the preoperative and postoperative IIEF scores of the patients in this study.

**Table 3:** Preoperative and postoperative IIEF scores of the patients

IIEF	Preoperative	Postoperative			*P*-value^a^
		3 months	6 months	12 months	
Total	23.2 ± 2.9	41.8 ± 4.5	55.3 ± 6.2	61.9 ± 9.5	< 0.05
Erectile function domain	6.5 ± 2.5	15.2 ± 3.4	20.5 ± 3.2	25.5 ± 3.9	< 0.05
Satisfaction domain	6.4 ± 2.4	12.8 ± 1.1	14.5 ± 2.2	14.9 ± 4.5	< 0.05

*IIEF* International Index of Erectile Function

^a^The preoperative IIEF scores and all of the follow-up period (postoperative 3, 6, and 12 months) scores were compared, and statistical significance was observed

**Fig. 1:**
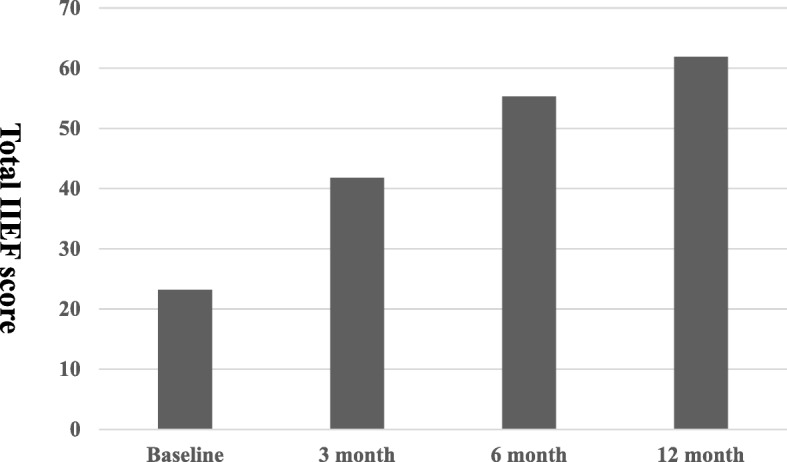
Total IIEF score. Statistically significant difference was observed for baseline versus all postoperative parameters

**Fig. 2:**
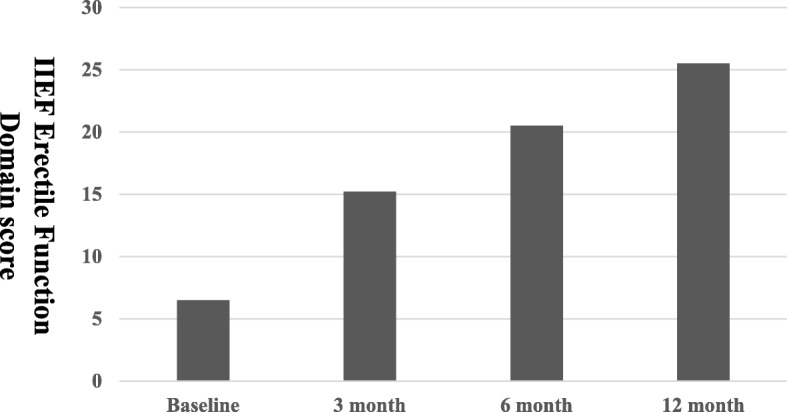
IIEF erectile function domain scores. Statistically significant difference was observed for baseline versus all postoperative parameters

**Fig. 3:**
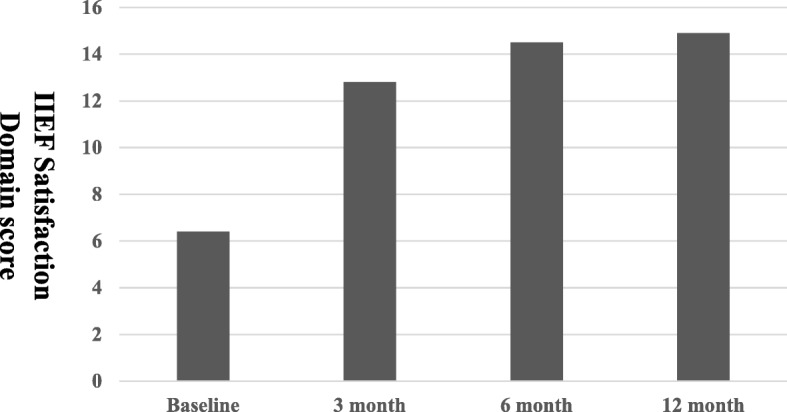
IIEF satisfaction domain scores. Statistically significant difference was observed for baseline versus all postoperative parameters

The total mean preoperative IIEF scores, IIEF erectile function domain scores, and IIEF satisfaction domain scores were 23.2 ± 2.9, 6.5 ± 2.5, and 6.4 ± 2.4, respectively. Postoperatively, the total IIEF scores were 41.8 ± 4.5, 55.3 ± 6.2, and 61.9 ± 9.5 at 3, 6, and 12 months, respectively (Fig. 1). The IIEF erectile domain scores were 15.2 ± 3.4, 20.5 ± 3.2, and 25.5 ± 3.9 at 3, 6, and 12 months, respectively (Fig. 2). Furthermore, the IIEF satisfaction domain scores were 12.8 ± 1.1, 14.5 ± 2.2, and 14.9 ± 4.5 at 3, 6, and 12 months, respectively (Fig. 3). Statistically significant improvements were observed for all postoperative total IIEF scores (*P* < 0.05), all postoperative IIEF erectile domain scores (P < 0.05), and all postoperative IIEF satisfaction domain scores (P < 0.05), compared with the preoperative scores (Table 3).

## Discussion

IPP implantation is a well-known, established, and considerably satisfactory treatment option for ED. Although an IPP is regarded to have good mechanical reliability in the long term, patient satisfaction with penile length after surgery has not been achieved because of the patient’s expectation in relation to the preimplantation erect penis length. The causes of dissatisfaction with the penile length are long ED duration, obesity, in which there are added tissues in the prepubic area, and acquired penile disorder such as Peyronie’s disease [7]. Deveci et al. [8] reported the stretched flaccid penile length before and after two- or three-piece IPP implantation in 56 patients. Although there were no statistically significant differences between the preoperative and postoperative penile lengths, 72% of the patients complained of a decrease in penile length after surgery. However, one study comparing the erect penile length after IPP implantation, through induced intracavernosal injection of vasoactive agents, with the length before surgery found that all 11 patients experienced a decrease in erect penile length, from 0.2 to 3.0 cm, after IPP implantation. Furthermore, 45.5% of the recipients reported that their subjective penile length shortened after surgery, and no patient thought that his postoperative penile length was longer than its preoperative length [9]. Henry et al. [10] prospectively assessed penile length measurements for 1 year after IPP implantation with the Coloplast Titan (Coloplast, Minneapolis, MN, USA), using an aggressive new length measurement technique to overcome reduced penile length. These patients underwent daily inflation of the IPP for 6 months and maximal inflation for 1–2 h daily for 6–12 months. Of these patients, 64.5% were satisfied with their penile length at 1 year and 74.2% of patients reported that their penile length increased. Because of the aggressive cylinder sizing and postoperative penile rehabilitation inflation protocol, all of the patients, except for two, experienced an increase (approximately 1 cm) in the stretched penile length. To prevent penile shortening, concomitant surgical interventions, such as a sliding technique, suprapubic lipectomy, suspensory ligament release, and ventral phalloplasty, have been developed and used [11, 12].

Although the exact mechanism of penile length shortening after IPP implantation has not been fully elucidated, incorrectly measuring the corporeal length during surgery and lack of glans tumescence after implantation might decrease the penile length [9]. Intraurethral alprostadil injection or oral phosphodiesterase type 5 inhibitors are used for enhancing the soft glans after penile prosthesis implantation to increase the length of a shortened penis [13, 14].

In the 1990s, the AMS 700 Ultrex penile prosthesis was developed to prevent penile shortening and increase penile girth and length. Montague et al. [3] reported that 50 patients underwent implantation using the AMS 700 Ultrex. The penile length was increased by 1 cm in 12 patients, and in 28 patients, the postoperative penile length was the same as the intraoperative length expansion. However, because S-shaped deformities have occurred when using the AMS 700 Ultrex prosthesis and its durability is low, urologists have been discouraged from using this IPP. AMS 700 LGX, which allows both girth and length expansion of the cylinders with up to 20% elongation, was developed to overcome these disadvantages. Recently, one prospective study reported the mean penile length and recipient satisfaction after AMS 700 LGX implantation [5]. At 6 and 12 months, the stretched flaccid penile length was longer than the preoperative length. Six and 12 months postoperatively, the IIEF desired domain scores and overall satisfaction scores were statistically different from the preoperative scores. However, the mean Erectile Dysfunction Inventory of Treatment Satisfaction scores did not show significant improvement after implantation.

In the present study, at 6 and 12 months postoperatively, the stretched penile length was longer than the preoperative length. We confirmed that all postoperative total IIEF scores, postoperative IIEF erectile function domain scores, and postoperative satisfaction domain scores significantly improved, compared with the preoperative scores. Furthermore, complications such as infection, skin necrosis, and erosion occurred rarely. The mechanical malfunction rate was also very low, and no mechanical complications such as a cylinder aneurysm or S-shaped deformity occurred. Therefore, the AMS 700 LGX is an excellent IPP to preserve penile length in patients who are concerned about decreases in their original penile length and has a low rate of complications and mechanical malfunction.

The present study has several limitations. First, this is a retrospective study. Second, the IPP implantation procedures were performed by an andrologist who performs a high volume of these procedures; therefore, it is difficult to generalize our results to surgeons who are less experienced. Third, this study excluded patients with Peyronie’s disease and corporal fibrosis as well as those who underwent a reoperation. A further study about the usefulness of AMS 700 LGX in patients with these conditions should be performed. Fourth, there are no validated questionnaires for evaluating erection and patient satisfaction with the IPP; hence, we used the IIEF to evaluate patients’ satisfaction. Thus, an IPP-specific validated questionnaire should be developed in the future. Finally, we did not record the patients’ weight, which could have resulted in some differences in the stretched flaccid penile lengths. Despite these limitations, our present study provides data on the outcomes of the use of and patient satisfaction with the AMS 700 LGX penile prosthesis in Korea for the first time.

## Conclusions

We believe that the AMS 700 LGX is a safe and reliable prosthesis for patients with ED and is effective in preventing penile shortening in patients undergoing IPP implantation, with high patient satisfaction. A longer follow-up of these patients and a prospective study are mandatory to confirm these results.

## Abbreviations

AMS: American Medical Service
ED: Erectile dysfunction
IIEF: International Index of Erectile Function
IPP: Inflatable penile prosthesis
